# Association between periodontal status and degree of maxillary sinus mucosal thickening: a retrospective CBCT study

**DOI:** 10.1186/s12903-021-01737-3

**Published:** 2021-08-11

**Authors:** Tianyi Zhang, Zhengquan He, Huan Tian

**Affiliations:** 1 School of Stomatology, Shanxi Medical University, 56th Xinjian South Road, Taiyuan, 030001 China; 2 Department of Orthodontics, Changsha Stomatological Hospital, Hunan University of Traditional Chinese Medicine, 389th Youyi Road, Changsha, 410004 China

**Keywords:** Cone-beam computed tomography, Mucosal thickening, Periodontitis, Minimum residual alveolar bone height

## Abstract

**Background:**

It is well known that periodontitis can stimulate thickening of the maxillary sinus mucosa, but the association between periodontitis status and the degree of maxillary sinus mucosal thickening (maxMT) has not been reported. The objectives of this study were to investigate the effect of periodontal status of maxillary molars on the degree of maxMT.

**Methods:**

Retrospective analysis of cone-beam computed tomographic (CBCT) images of 203 periodontitis cases with maxMT. Parameters related to periodontitis in maxillary molars were measured and recorded on CBCT images. The dimension and length of the maxMT were also recorded. Multiple linear regression analysis was used to identify periodontal factors influencing the severity of maxMT, and multivariate logistic regression analysis was used to identify the odds ratio of these factors.

**Results:**

The factors affecting the degree of maxMT were mainly the amount of alveolar bone loss (ABL) and the minimum residual alveolar bone height (miniRABH). Compared to mild ABL, severe and moderate ABL were more likely to display severe maxMT. And the lower the miniRABH, the more severe the maxMT.

**Conclusions:**

The severity of periodontal status of maxillary molars can influence the degree of maxMT.

## Background

Periodontitis is an inflammatory disease in which plaque microorganisms are the initiating factor, following the formation of dental plaque and causing the destruction of the supporting tissues of the periodontium [1]. It is a disease with a high prevalence, up to 90% in adults and up to 11% severe cases among them and is the leading cause of tooth loss in people [2]. Periodontitis not only causes damage to the local tissues of the periodontium, but may also affect the health of adjacent tissues and even the whole system [3, 4].

More literature [5, 6] reports that periodontitis in the maxillary molar region can stimulate thickening of the maxillary sinus mucosa, with 32% of maxillary posterior periodontitis associated with maxillary sinus mucosal thickening (maxMT). These studies investigated the frequency of maxMT in different conditions such as alveolar bone resorption (ABL), minimum residual alveolar bone height (miniRABH) and root furcation lesions in periodontitis, but the relationship between the degree of maxMT and the status of periodontitis has not been analysed [5, 7, 8]. The status of the maxillary sinus mucosa is extremely important for internal and external maxillary sinus floor elevation. Significant thickening of the mucosa and an inflammatory state of the maxillary sinus can cause perforation of the mucosa, bleeding and aggravation of inflammatory state in maxillary sinus floor elevation, which is the main reason for the failure of the maxillary sinus floor elevation operation [9]. Manor et al. found through a clinical study that most patients who developed postoperative sinusitis had a history of preoperative sinusitis and maxMT, and despite certain preoperative therapeutic measures, there was still a risk of increased sinus inflammation after surgery [10].

Cone-beam computed tomographic (CBCT), as a common dental examination equipment, can clearly show the structure of the teeth, periodontal tissues and jaw bones [11, 12]. At the same time, because of its advantages of low radiation dose, high resolution and clear details of the maxillary sinus, it is widely used in the examination of maxillary sinus mucosa [13]. CBCT images can more accurately show the thickening of the mucosa, inflammation and cyst fluid level rise [6, 14]. Therefore,the use of CBCT in oral implantology to analyse the relationship between the maxillary posterior alveolar bone and the maxillary sinus is of great advantage.

There are more analyses in the current literature on the possibility and frequency of periodontitis affecting maxMT by using CBCT images [5, 13], but what periodontitis status are associated with the degree of maxMT are less frequently reported. In this study, the above problems were discussed by retrospective analysis of the images data of CBCT.

## Methods

This study was approved by the Scientific Research Committee of Changsha Stomatological Hospital (CKY2020-0605) and conducted in strict accordance with the Declaration of Helsink (2013). Necessary measures have been taken to protect patient privacy during the collection of clinical and imaging data.

## Research data

The images of periodontitis patients with maxMT who visited the department of periodontology between January 2015 and September 2020 were selected for analysis. The records of periodontal indexes such as periodontal pocket depth and clinical attachment loss were also reviewed to confirm that the periodontitis was clinically confirmed.

Inclusion criteria: (1) The patient's age were over 18 years [15]; (2) The CBCT images were clear and could show the anatomical structure of the maxillary sinus and related teeth completely, with no missing of posterior maxillary teeth. CBCT (Sirona, Germany) images related parameters: voltage 85 kv, current 6 mA, exposure time 14.4 s, slice thickness 0.3 mm, field volume 11 cm*10 cm. According to Cagici et al. we used > 2 mm as the diagnostic criterion for maxMT [16]. Diagnostic criteria for periodontitis images: resorption of alveolar bone beyond the cementum–enamel junction(CEJ) by more than 2 mm [17].

Exclusion criteria: (1) acute inflammation of the maxillary sinus with elevated fluid level and cysts in the sinus; (2) implants in the posterior maxilla; (3) periapical lesions and caries, fractures, root canal therapy or previous periodontal therapy and loss of maxillary posterior teeth (except for missing maxillary third molars); (4) in order to compare the classification of root furcation lesions, the cases of fusion root of maxillary second molars were excluded. The presence of these factors could also lead to thickening of the maxillary sinus mucosa and were confounding factors, making it difficult to determine whether the source of the thickening of the maxillary sinus mucosa was periodontitis or these factors.

**Fig. 1:**
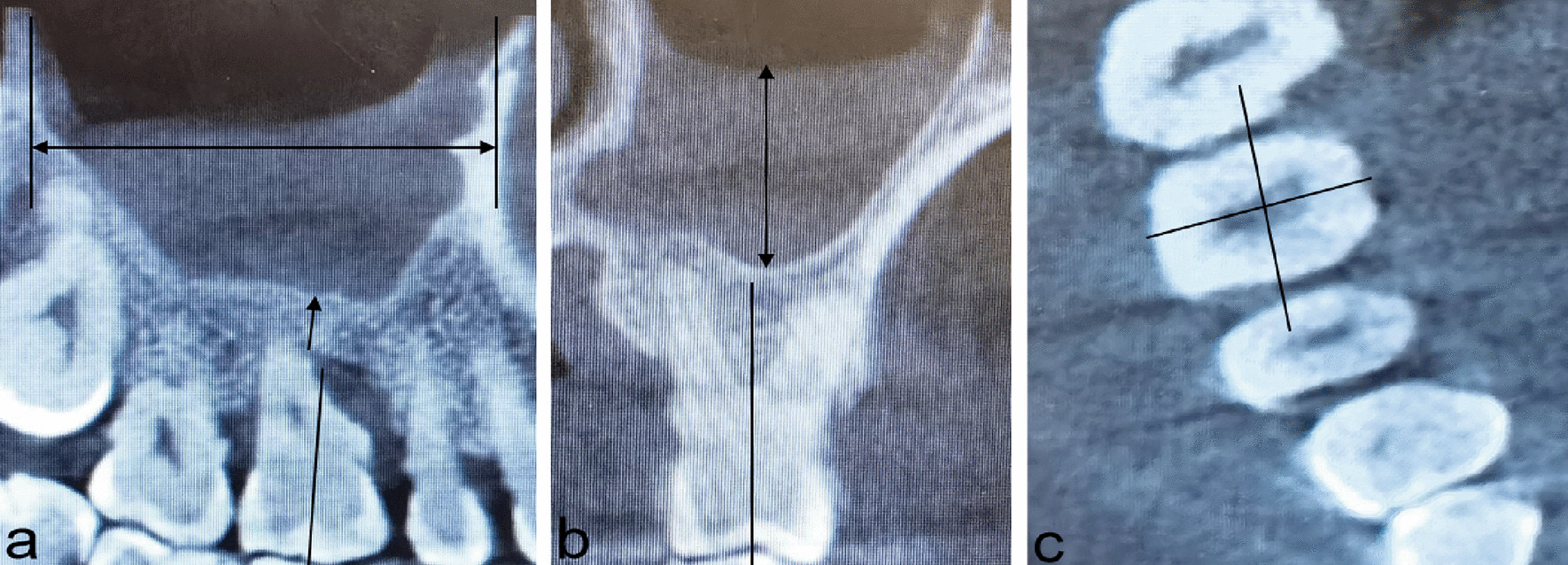
Three-dimensional reconstruction image of maxMT. **a** Sagittal view, double-ended arrow: the length of maxMT; Single-ended arrow: the minRABH. **b** Coronal view, double-ended arrow: the thickness of the mucosa of the maxillary sinus. **c** Horizontal view. Abbreviations: maxMT maxillary sinus mucosal thickening; miniRABH minimum residual alveolar bone height.

Measurements: coronal and sagittal: through the long axis of the tooth; horizontal: through the center of the molar pulp cavity (Fig. 1). The relevant measured value of the teeth with the most severe periodontal disease were analyzed.

### Measurement index

1. Maximum dimension and length of mucosal thickening: The dimension of maxMT can be divided into three grades: mild (2–4 mm), moderate (> 4 to ≤ 10 mm) and severe (> 10 mm) [5]. The distance between the boundary of mucosal thickening was measured from the coronal plane and sagittal plane of CBCT, and the maximum value was recorded as the maximum length of mucosal thickening.
2. Root furcation lesion: the amount of bone resorption at the root furcation was measured from the horizontal section and classified into four degrees according to hamp's criteria. Class 0, no bone resorption; Class 1 bone resorption ≤ 3 mm; Class 2 bone resorption > 3 mm, no penetration; Class 3 penetration root furcation [18, 19].
3. Vertical infrabony defects were graded as follows: type I, no vertical infrabony defects; type II, not more than middle thirds of root; type III, infrabony defects extending to the apical third of the tooth [20].
4. Minimum residual bone height: minimum distance from the apical of the lesion to the floor of the maxillary sinus.These values were classified into three levels. Level 1 was < 4 mm, Level 2 was 4–10 mm, and Level 3 was ≥ 10 mm [21].
5. ABL: the amount of ABL were measured from mesia and distal of the maxlliary molars as well as the buccal and lingual side. Normal alveolar bone height was within 2 mm of the boundary of CEJ. The percentage of maximal ABL relative to normal alveolar bone height was calculated. Bone resorption less than 25% was considered to be mild, 25–50% was moderate and greater than 51% was severe [8].

The relevant test data were measured by two periodontists who had previously undergone training and were familiar with the criteria for measurement, and the images of 10 cases were randomly selected and measured twice with an interval of 2 weeks to test the reliability between the two measurers.The range of ICC value was 0.838–0.925, and the ICC value of the self-reliability was 0.958–0.972. The measured data were reliable and the error could be ignored.

### Statistical analyses

SPSS 26.0 statistical software was used for statistical analysis. The data were described by frequency, mean ± SD and range. Inter-measurer agreement was tested using group ICC coefficients. The periodontal parameters associated with maxMT were tested by using the Chi-square test and Fisher's exact test, and multiple linear regression analysis was used to identify periodontal factors related to the degree of maxMT. Multivariate logistic regression analysis was used to check the effect of changes in relevant factors on the degree of maxMT. *p* < 0.05 was considered statistically significant.

## Results

In this study, CBCT images of 203 periodontitis patients with maxMT were analyzed, including 114 males and 89 females aged 54.1 ± 11.8 years (23–85 years). The range of mucosal thickening was 8.25 ± 4.36 mm (2–16.6 mm). The corresponding basic distribution according to the parameters could be seen in the Table 1. And it could be seen that the patients who were seen in the periodontiology department were relatively older and had a relatively poor periodontal condition. Type II and type III vertical infrabony defectss also accounted for the majority with 39.9% and 36.5%, respectively, while type I accounted for only 23%, and root furcation lesions accounted for 55.2% of all cases. The percentage of ABL from mild to severe was 49.8%, 34.0% and 16.3%, respectively. The Chi-square test and Fisher's exact test showed that the degree distribution of miniRABH and ABL on different grades of maxMT was significant different.

**Table 1:** Relationship between periodontal-related parameters and the degree of maxMT

			*Mucosal thickening*			
Parameters	n		2–4	> 4 to ≤ 10	> 10 mm	*p*
Gender						0.251
Male	114 (56.2%)		14 (6.9%)	53 (26.1%)	47 (23.2%)	
Female	89 (43.8%)		15 (7.4%)	47 (23.2%)	27 (13.3%)	
Age						0.139
< 44	44 (21.7%)		2 (1.0%)	24 (11.8%)	18 (8.9%)	
44–64	114 (56.2%)		19 (9.4%)	51 (25.1%)	44 (21.7%)	
≥ 65	45 (22.2%)		8 (3.9%)	25 (12.3%)	12 (5.9%)	
Molar site						0.572
First molar	127 (62.6%)		18 (8.9%)	66 (32.5%)	43 (21.2%)	
Second molar	76 (37.4%)		11 (5.4%)	34 (16.7%)	31 (15.3%)	
Furcation lesion						0.038
Calss 0	91 (44.8%)		13 (6.4%)	52 (25.6%)	26 (12.85%)	
Class I	34 (16.7%)		5 (2.5%)	12 (5.9%)	17 (8.4%)	
Class II	49 (24.1%)		9 (4.4%)	26 (12.8%)	14 (6.9%)	
Class III	29 (14.3%)		2 (1.0%)	10 (4.9%)	17 (8.4%)	
Vertical infro-bony defects						0.863
Type I	48 (23.6%)		8 (3.9%)	25 (12.3)	15 (7.4%)	
Type II	81 (39.9%)		12 (5.9%)	40 (19.7%)	29 (14.3%)	
Type III	74 (36.5%)		9 (4.4%)	35 (17.2%)	30 (14.8%)	
MinRABH						0.031*
< 4	96 (47.3%)		9 (4.4%)	42 (20.7%)	45 (22.2%)	
4–10	91 (44.8%)		17 (8.4%)	48 (23.6%)	26 (12.8%)	
≥ 10	16 (7.9%)		3 (1.5%)	10 (4.9%)	3 (1.5%)	
ABL						0.000***
Mild	101 (49.8%)		23 (11.3%)	55 (27.1%)	23 (11.3%)	
Moderate	69 (34.0%)		4 (2.0%)	32 (15.8%)	33 (16.3%)	
Severe	33 (16.3%)		2 (1.0%)	13 (6.4%)	18 (8.9%)	

Chi-square test and Fisher's exact test

*maxMT* maxillary sinus mucosal thickening, *minRABH* minimum residual alveolar bone height, *ABL* alveolar bone loss

*p < 0.05, ***p < 0.001

Multiple linear regression revealed that ABL and miniRABH were the main factors significantly influencing the dimension and length of maxMT (Tables 2, 3). Further multivariate logistic regression analysis disclosed the odds ratio (OR) of ABL and miniRABH affecting the severity of maxMT (with mild maxMT as a reference). Compared to mild ABL, moderate and severe ABL were more likely to display more severe maxMT. As the degree of ABL increases, the OR values increased from 3.894 (95% CI, 0.683–15.156, *p* = 0.126) to 4.209 (95% CI, 1.169–22.243, *p* = 0.028) for moderate maxMT and from 12.071 (95% CI, 3.319–46.416, *p* = 0.000) to 18.290 (95% CI, 2.857–117.094, *p* = 0.002) for severe maxMT. On the contrary, as miniRABH decreases, the OR values increased from 1.301 (95% CI, 0.268–6.039, *p* = 0.744) to 2.541 (95% CI, 0.435–14.863, *p* = 0.301) for moderate maxMT and from 4.567 (95% CI, 0.629–33.160, *p* = 0.133) to 17.008 (95% CI, 2.023–142.981, *p* = 0.009) for severe maxMT. Statistics showed the greatest likelihood of triggering severe maxMT when miniRABH < 4 mm (Table 4).

**Table 2:** Periodontal parameters associated with the dimension of maxMT

Parameters	Dimension of maxMT		
	Coefficient	T	*p*
Gender	− 0.119	− 1.755	0.081
Age	− 0.092	− 1.361	0.175
Molar site	0.014	0.206	0.837
Furcation lesion	− 0.046	− 0.579	0.563
Vertical infrabony defects	− 0.094	− 1.179	0.240
MinRABH	− 0.212	− 2.841	0.005**
ABL	0.295	4.028	0.000***

Multiple linear regression analysis

*maxMT* maxillary sinus mucosal thickening, *minRABH* minimum residual alveolar bone height, *ABL* alveolar bone loss

**p < 0.01, ***p < 0.001

**Table 3:** Periodontal parameters associated with the length of maxMT

Parameters	Length of maxMT		
	Coefficient	T	P
Gender	− 0.200	− 2.977	0.003**
Age	0.010	0.144	0.886
Molar site	0.031	0.467	0.641
Furcation lesion	− 0.129	− 1.628	0.105
Vertical infrobony defects	0.097	1.220	0.224
MinRABH	− 0.216	− 2.907	0.004**
ABL	0.208	2.858	0.005**

Multiple linear regression analysis

*maxMT* maxillary sinus mucosal thickening, *minRABH* minimum residual alveolar bone height, *ABL* alveolar bone loss

**p < 0.01

**Table 4:** Odds Ratio (OR) and 95% Confidence Interval (CI) for the risk of severe maxMT

Parameters	maxMT (>4 to ≤10 mm vs 2–4 mm)			maxMT (> 10 mm vs 2–4 mm)		
	OR	95% CI	*p*	OR	95% CI	*p*
MiniRARH						
4–10 mm vs ≥ 10 mm	1.301	0.268–6.309	0.744	4.567	0.629–33.160	0.133
< 4 mm vs ≥ 10 mm	2.541	0.435–14.863	0.301	17.008	2.023–142.981	0.009**
ABL						
Moderate vs mild	3.894	0.683–15.156	0.126	12.071	3.319–46.416	0.000***
Severe vs mild	4.209	1.169–22.243	0.028*	18.290	2.857–117.094	0.002**

Statistics: multivariate logistic regression analysis

*maxMT* maxillary sinus mucosal thickening, *minRABH* minimum residual alveolar bone height, *ABL* alveolar bone loss, *miniRABH* minimum residual alveolar bone height

*p < 0.05, **p < 0.01, ***p < 0.001.

## Discussion

In this study, the incidence of periodontitis was mainly focused on the age above 45 years, which was consistent with the peak age of onset of periodontitis in relevant epidemiological studies [22].

The relevant factors associated with maxMT are mainly the amount of ABL, vertical infrobony pockets, minRABH and the classification of root furcation lesions in previous studies [5]. And some studies have analyzed only the minRABH and the amount of ABL associated with maxMT [19–22]. And this is also the factors that influences the degree of maxMT in our study. The attachment loss of vertical infrobony pockets as well as root furcation lesions in our study did not correlate with the degree of the maxMT, similar to the results of Zhang et al. [15]. The results shows that the amount of ABL is a hallmark of the severity of periodontitis and is one of the ultimate outcomes of periodontitis [23]. As the amount of ABL increases, the closer the lesion to the floor of the maxillary sinus, the more severe the maxMT. Periodontitis not only causes alveolar bone loss but also stimulates the thickening of the maxillary sinus mucosa in the posterior maxillary area, which has a significant impact on the implant placement and elevation of the maxillary sinus mucosa. Thus the results embody the necessity of periodontal treatment and early intervention to alleviate periodontal symptoms which is necessary for the health of the adjacent tissues.

The relevant mechanisms by which periodontitis affects the maxMT are as follows: periodontitis can affect the maxillary sinus mucosa by local inflammatory reactions as well as by bacterial invasion. Relevant anatomical study has shown that the floor of the maxillary sinus is not a flat layer of cortical bone and small microporosities are present at intervals [24]. There are also more pores in the alveolar bone, which should be fundamental evidence of the spread of periodontal inflammation into the maxillary sinus. In the present study, we also found more upper posterior teeth with roots in direct contact with the mucosa of the maxillary sinus floor, separated only by a thin bone plate, or even protruding directly into the maxillary sinus.This is also supported by relevant imaging studies of CBCT [11]. Therefore, inflammation of the periodontium can easily affect the health of the mucosa of the maxillary sinus. The same periodontal pathogenic bacteria such as *Fusobacterium nucleatum* and *Prevotella intermedia* can be detected in some maxillary lesions and in maxillary sinus lesions, which confirms that periodontal pathogenic bacteria can enter the maxillary sinus through the alveolar bone and cause an inflammatory response in the mucosa of the maxillary sinus [25].

Clinical examination indexes such as periodontal pocket depth and attachment loss were not included in this study, mainly because this was a retrospective study and it was difficult to ensure that previous clinical examinations were performed under the same criteria. Further, these clinical examination indexes can also be partially represented on images. And it is very necessary to remove the duplication in statistics, otherwise it will have some negative impact on the results. When conducting prospective studies, these clinical indexes can be included.

The present study also has some limitations. Although some consistency testing has been performed, errors in experimental measurements and limitations in the selection of experimenters still exist. It will also have a minor negative impact on the conclusions. If there are errors in the measurement of some critical values, this may lead to differences in grading of periodontal indexes and therefore the intensity of the effect of different status of periodontitis on the degree of maxMT may be different. Therefore, more rigorous experiments are necessary to determine the exact effect of these factors. Prospective studies can be carried out to analyse the relationship between periodontitis and maxMT in conjunction with the patient's clinical examination and CBCT.

## Conclusions

With the progression of periodontitis towards and closer to the maxillary sinus floor, the maxMT becomes more severe and early intervention of periodontitis is also necessary for periodontal health and the health of the maxillary sinus mucosa.

## Data Availability

Data are available from the corresponding authors upon reasonable request.

## Abbreviations

maxMT: Maxillary sinus mucosal thickening
CBCT: Cone-beam computed tomographic
OR: Odds ratio
ABL: Alveolar bone loss
miniRABH: Minimum residual alveolar bone height
CEJ: Cementum–enamel junction
